# Maternal benzo[a]pyrene exposure during critical gestational periods impairs offspring neurological development in rats: a mechanistic study of the Wnt/β-catenin signaling pathway

**DOI:** 10.3389/fnbeh.2025.1571122

**Published:** 2025-04-24

**Authors:** Nan Zhang, Nan Bo, Yuanhao Wang, Wenlin Bai, Wen Sun, Zhenlin Zhao, Yuanbao Zhang, Yingying Zhang, Lijian Lei, Jianjun Zhou, Wenping Zhang

**Affiliations:** ^1^Department of Toxicology, School of Public Health, Shanxi Medical University, Taiyuan, China; ^2^Department of Children and Adolescences Health, School of Public Health, Taiyuan, China; ^3^Department of Epidemiology, School of Public Health, Shanxi Medical University, Taiyuan, China; ^4^Shenzhen Ruipuxun Academy for Stem Cell and Regenerative Medicine, Shenzhen, China; ^5^Beijing Key Laboratory of Occupational Safety and Health, Institute of Urban Safety and Environmental Science, Beijing Academy of Science and Technology, Beijing, China; ^6^MOE Key Laboratory of Coal Environmental Pathogenicity and Prevention, Shanxi Medical University, Taiyuan, China; ^7^Research Centre of Environmental Pollution and Major Chronic Diseases Epidemiology, Shanxi Medical University, Taiyuan, China; ^8^Research Center for Translational Medicine, Cancer Stem Cell Institute, East Hospital, Tongji University School of Medicine, Shanghai, China; ^9^Key Laboratory of Physiology, Taiyuan, China

**Keywords:** B[a]P, mid-gestation, developmental landmarks, learning and memory function, LiCl

## Abstract

**Introduction:**

Mid-gestation is a critical period for the development of the nervous system. Exposure to exogenous harmful chemicals during this period may lead to longterm neurological developmental abnormalities in offspring. Benzo[a]pyrene (B[a]P) is a commonly occurring neurotoxic environmental pollutant that can pass through the placental barrier and blood-brain barrier (BBB), thereby affecting placental nerve development.

**Methods:**

To investigate the neurotoxic mechanism of B[a]P on offspring exposed in mid-gestation, pregnant rats were exposed to B[a]P (25 mg/kg) from gestation days 8 to 14. Meanwhile, as an agonist of Wnt/β-catenin signaling pathway, lithium chloride (LiCl) was administered to observe the intervention effects.

**Results:**

The results showed that in rats exposed to B[a]P in mid-gestation, the developmental nodes of the offspring were delayed, and the neurosensory sensitivity of the offspring was reduced. These offspring also had cognitive impairments in adulthood. Subsequent morphological and protein experiments showed that the exposed offspring had reduced neuronal complexity in the CA1 region of the hippocampus, decreased β-catenin expression, and increased GSK-3β expression in the hippocampal tissue. However, all these indexes can be reversed by LiCl.

**Discussion:**

These results suggest that B[a]P exposed in mid-gestation pregnancy may lead to neurological damage in the offspring by downregulating the Wnt/β-catenin signaling pathway.

## Highlights

- Exposure to benzo[a]pyrene during mid-gestation can disrupt early offspring development.
- Exposure to benzo[a]pyrene during mid-gestation can lead to damage in the learning and memory function of adult offspring.
- Downregulation of the Wnt/β-catenin signaling pathway is relevant to the neurological damage in adult offspring caused by mid-gestation exposure to benzo[a]pyrene.

## 1 Introduction

Polycyclic aromatic hydrocarbons (PAHs) widely exist in the environment and have carcinogenicity, teratogenicity, and mutagenicity (Abdel-Shafy and Mansour, 2016; Meng et al., 2021; Bukowska et al., 2022). Being an important component of PAHs, benzo[a]pyrene (B[a]P) is an important indicator of PAHs and is often used to represent the environmental and health risks of the entire PAH population (Peng et al., 2023). However, the damaging effect of B[a]P on the nervous system remains to be studied. B[a]P has high liposolubility and can easily enter the fetal blood system through the placenta and then act on the fetal brain nerve tissue through the blood–brain barrier (BBB) (Al-Saleh et al., 2013).

The occurrence of adult diseases is closely related to the adverse factors experienced during early development of life (fetal period and infant period) (Arima and Fukuoka, 2020). Many studies have evaluated the impairment of neuromotor function, learning, and memory in animals and humans exposed to B[a]P postnatally. The study by Bouayed et al. (2009) showed that there were different behavioral disorders during postnatal development and infancy in young rats exposed to B[a]P during lactation. Chen et al. (2012) reported that the behavioral damage caused by exposure to B[a]P after birth may last for a long time, which may not be obvious during adolescence but exists in young adulthood. Das et al. (2019) found that adolescent B[a]P exposure causes learning and memory impairment and neuronal damage in adulthood. Pregnancy is a long period of time, and each organ has its corresponding window of development. Capturing the critical window of fetal neurodevelopment is essential for the prevention and control of neurological disorders.

The proliferation and migration of neural cells in the fetal brain start to accelerate in mid-gestation, reaching a peak shortly thereafter (Iruretagoyena et al., 2014). In addition, mid-gestation is also the beginning of the synaptic formation period and lasts several years after birth (Nadarajah and Parnavelas, 2002). During the critical period of nervous system growth and development, fetal exposure to exogenous chemicals not only increases the risk of adverse birth outcomes but also leads to long-term potential neurological damage. Two parallel studies in Korea and Norway reported that an increase in B[a]P content in maternal diet during pregnancy may lead to a decrease in fetal birth weight and head circumference (Duarte-Salles et al., 2013; Lamichhane et al., 2016). In animal experiments, exposure to BAP was also found to increase the preterm birth rate, reduce the birth weight of pregnant rats and offspring, and increase placental weight (Zhao et al., 2024). Furthermore, exposure to PAHs may hurt children’s IQ and learning ability (Kalia et al., 2017). However, few studies have directly addressed neurotoxicity due to B[a]P exposure during critical periods of brain development. Therefore, investigating the neurotoxicity and its possible mechanisms of B[a]P exposure during mid-gestation is our main focus in this study.

Neurological reflex tests in early postnatal life can determine if there are abnormalities in the visual and vestibular systems and limb pressure or tactile receptors of the offspring. Additionally, behavioral tests are often used to assess damage to learning and memory in animals. For instance, the novel object recognition test evaluates learning and memory by assessing the offspring’s memory for novel objects (Chao et al., 2022). Meanwhile, the Morris water maze test assesses spatial memory by recording the time it takes for an animal to find a platform (Liu and Borisyuk, 2024). Subsequently, the organizational basis of learning and memory function impairment was verified by examining the growth structure of neurons in the hippocampal CA1 region. Meanwhile, the potential mechanism of benzo(a)pyrene-induced learning and memory impairment was explored by measuring the levels of key proteins in the hippocampus (Pedrazzoli et al., 2021).

The Wnt signaling pathway is an important regulatory pathway for neuronal proliferation and development. In a typical Wnt signaling pathway, activation of disheveled (Dvl) proteins will initiate transcription of Wnt target genes by inhibiting glycogen synthase kinase-3 (Gsk-3) activity, which will translocate β-catenin proteins to the nucleus and bind to T-cell factor/lymphoid enhancers (Oliva et al., 2013). Moreover, exposure to benzo[a]pyrene may lead to an increase in the phosphorylation and degradation complex of GSK-3β in the cytoplasm, which in turn downregulates the Wnt/β-catenin pathway (Cerpa et al., 2011). LiCl, as a non-specific agonist of GSK-3β protein in the Wnt pathway (Liu et al., 2018), mimics the activation process of the Wnt/β-catenin signaling pathway.

Since Cade (1949) discovered lithium salts as a treatment for psychiatric diseases, lithium has been regarded as a gold standard for treating bipolar disorder and depression. Because lithium carbonate is strongly alkaline, to avoid burning the mouth and esophagus, we chose neutral lithium chloride (Chiou et al., 2021). It has been noted in the literature that there appears to be no difference in the pharmacokinetics between lithium chloride and lithium carbonate (Smith, 1976). Although there is controversy over the idea that lithium causes prenatal abnormalities, a prospective Israeli investigation found no difference in the incidence of congenital malformations between lithium-exposed and control groups (Diav-Citrin et al., 2014).

## 2 Materials and methods

### 2.1 Animals

Seventy-two Sprague-Dawley (SD) rats (10 weeks old, 36 males weighing 280 ± 20 g and 36 females weighing 240 ± 20 g) were purchased from the Experimental Animal Center of Shanxi Medical University. Animals were housed randomly for 12 h day/12 h night with an ambient temperature of 23–25°C, a humidity of 50%–55%, and free access to water and food. The experiment was approved by the Ethics Committee of Shanxi Medical University.

### 2.2 Treatments

After 1 week of training, SD rats were mated 1:1, and the first day of embolism was considered day 0 of gestation (GD0). Pregnant rats were randomly divided into the following nine groups (n = 6): control group (Ctl, treated with water), vehicle group (Vcl, treated with plant oil), B[a]P group (B[a]P, treated with 25 mg/kg B[a]P), LiCl low-dose intervention group (LL, treated with 25 mg/kg B[a]P and 10 mg/kg LiCl), LiCl medium-dose intervention group (LM, treated with 25 mg/kg B[a]P and 20 mg/kg LiCl), LiCl high-dose intervention group (LH, treated with 25 mg/kg B[a]P and 40 mg/kg LiCl), lithium chloride alone low dose group (LiCl-L, treated with 10 mg/kg LiCl), lithium chloride alone medium dose group (LiCl-M, treated with 20 mg/kg LiCl), and lithium chloride alone high dose group (LiCl-H, treated with 40 mg/kg LiCl).

During the 7 days of continuous intervention from GD8 to GD14, each intervention group of LiCl was first given an equal volume of 10, 20, and 40 mg/kg of LiCl solution by gavage, followed by 25 mg/kg of B[a]P solution by gavage after a 2-h interval. The health status of the animals was observed and recorded daily, and the date of natural birth of pregnant rats was recorded as postnatal day 0 (PND0), and the birth weight of the offspring was also recorded.

**Figure 1:**
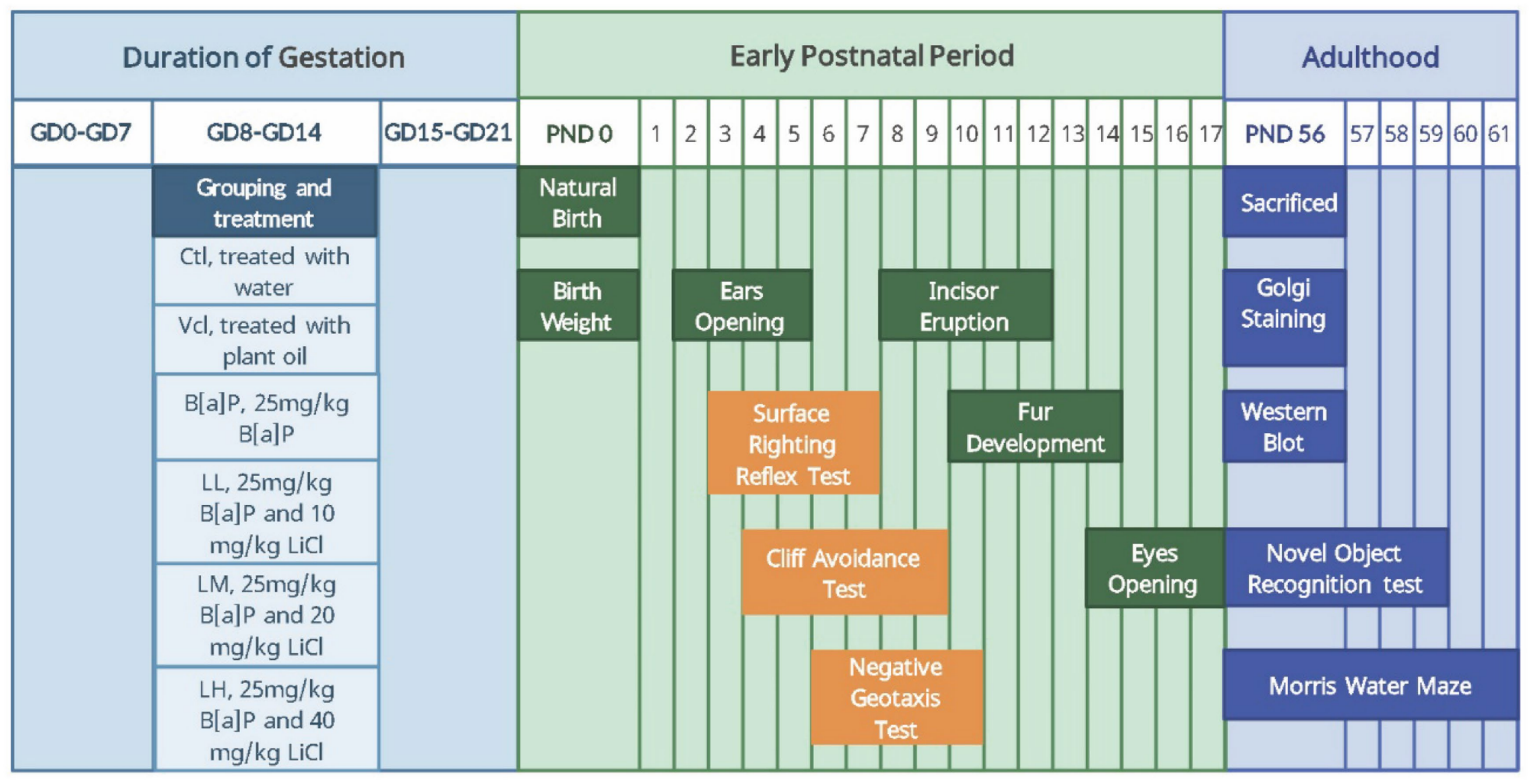
The time line of experimental arrangement. ♀5,5♂ from each litter were selected to test developmental landmarks and the neonatal sensory and motor development: Body weight (PND0), ears opening (PND2–5), incisor eruption (PND8–12), fur development (PND10–14), eye opening (PND14–17), surface righting reflex test (PND3–7), cliff avoidance test (PND4–9), negative geotaxis test (PND6–10). 2♀,2♂ from each litter were selected to test learning and memory: Novel Object Recognition (PND56–59), Morris-water maze (PND56–61). 2♀,2♂ from each litter were selected to be sacrificed (PND56) for molecular biological detection: Golgi staming and Western blot. In the early postnatal stage, we observed all offspring’s developmental milestones and conducted neurodevelopmental reflex tests. Some offspring were tested with the Morris water maze and others with the novel object recognition test after adulthood.

At the individual level, the developmental nodes and neural reflex functions of the offspring were assessed in the early postnatal period, and the episodic and spatial learning memory abilities of the offspring were assessed in adulthood; at the cellular level, the dendritic morphology of hippocampal neurons of the adult offspring was observed; and at the protein level, the expression levels of the key proteins GSK-3β and β-catenin in the Wnt/β-catenin pathway were determined in the adult offspring. The study design is shown in Figure 1. In the early postnatal stage, we observed all offspring’s developmental milestones and conducted neurodevelopmental reflex tests. Some offspring were tested with the Morris water maze, and others with the novel object recognition test after adulthood.

### 2.3 Developmental landmarks

During the period from PND2 to PND5, the time of complete opening of the auricles of both sides of the offspring was observed; during PND8 to PND12, the time of the upper incisors of the offspring was observed. During the PND10 to PND14, the time of uniform growth of abdominal hair and the absence of fleshy coloration was observed; and during PND14 to PND17, the time of complete disappearance of the ocular membranes in both eyes was recorded (Figure 1). Record the above time points and calculate the average developmental landmarks of each litter (Equation 1).


(1) $$DL=\frac{\sum_{i=1}^{n}\mathrm{time}\;\mathrm{point}}{n}$$

DL is the average time to reach the developmental landmarks of each litter. n denotes the number of offspring per litter.

### 2.4 Neurodevelopmental reflex tests

#### 2.4.1 Surface righting reflex test

The offspring rats were slowly brought out of the nest and rested on a heating cushion. The offspring rats were timed from being placed in the supine position and were scored as passing if all 4 ft touched the surface within 10 s; otherwise, they were scored as failing. Rats received three tests during the day and were scored as positive when all tests were passed. Each offspring was subjected to testing in the period PND3–PND7 between 09:00 h and 12:00 h.

#### 2.4.2 Cliff avoidance test

The head and front paws of the offspring rats were placed on the edge of a platform 20 cm above the ground, and the rats were recorded as positive if they backed up or turned inside the platform within 30 s; otherwise, they were recorded as negative. Offspring rats were tested once a day, between 09:00 and 12:00 h, for each rat, for PND4–PND9.

#### 2.4.3 Negative geotaxis test

Offspring rats are placed in a head-down orientation on a 30°inclined plane. Within 120 s, the offspring rat is rotated 180° to a head-up orientation, indicating a positive result. If the offspring rat climbed off the platform or did not move, it was recorded as negative. Offspring rats were tested once a day, between 09:00 and 12:00 h, for each rat, for PND6–PND10 (Figure 1).

The time points of the above positive events were recorded for each offspring rat, and the mean time to completion of the development of neuromotor reflex function was calculated for each litter (Equation 2).


(2) $$NRF=\frac{\sum_{i=1}^{n}\mathrm{time}\;\mathrm{point}}{n}$$

NRF indicates the mean time to completion of the development of the neuromotor reflex function and n denotes the number of offspring per litter.

### 2.5 Golgi staining

At PND56, three litters of offspring rats were randomly selected from each group, and then one male and one female rat were randomly selected from the selected litter to be killed (Figure 1). Their brains were dissected for Golgi staining in frozen slices of 150 μm thickness. Twenty individual neurons were randomly selected from the hippocampal CA1 region of each rat. Neuronal morphology was reconstructed in three dimensions using Image J software, and the total number of branches and dendritic length were measured. The complexity of the total dendritic tree was estimated using Sholl’s analysis (Sholl and Uttley, 1953).

### 2.6 Novel object recognition test

The experiment was divided into a training phase and a testing phase. During the training phase, the rats were removed from their cages and placed in the center of the open space (100 cm × 100 cm × 50 cm (L × W × H)) for 5 min of free exploration on 3 consecutive days, after which the animals were placed back in their original cages. After 5 min, the rats were placed in the center of the open space again to allow them to freely explore for 5 min, and at the end of the training, the rats were placed back in their cages. On day 4, the rat was placed in the open field with four identical objects and allowed to explore freely for 5 min. The rat was then placed back in its original cage for 5 min, and the familiar objects were replaced with new ones. The rats were again placed in the open space and allowed to explore freely for 5 min. The rats were tested with PND56–PND59 between 15:00 h and 22:00 h each. The experimental indicator was calculated as follows (Equation 3).


(3) $$\mathrm{Discrimination rate}=\frac{\begin{array}{l} \mathrm{time spent exploring}\;\\ \mathrm{the}\;\mathrm{new}\;\mathrm{object} \end{array}}{\begin{array}{l} \mathrm{total time spent}\;\\ \mathrm{exploring}\;\mathrm{all}\;\mathrm{objects} \end{array}}\times 100\%$$

### 2.7 Morris water maze

The Morris water maze (MWM), which was also divided into a training phase and a spatial exploration test, consists of a swimming pool (150 cm in diameter and 50 cm high) and a hidden escape platform (9 cm in diameter and 20 cm high). The pool is filled with water, which is kept at a temperature of 23 ± 2°C. The entire palace body was divided into four quadrants: the southeast, northeast, southwest, and northwest quadrants, and the escape platform was fixed 1 cm below the water surface in the southwest quadrant throughout MWM training. During the training phase, the rats were placed in the water with their heads facing the inner wall in each of the four quadrants and swam freely to find the hidden platform within 60 s. If the rat could not find the platform within 60 s, it was artificially guided to the platform and held for 10 s, with a latency period of 60 s to find the platform. Each rat was placed in the water from four directions four times a day at 30-min intervals for 5 days. On day 6, the spatial exploration test was performed by gently placing the rat into the water, facing the inner wall from the northeast, and recording its exploration for 1 min in the swimming pool. The latency to find the platform and the number of crosses on the platform were recorded. The rats were tested with PND56–PND61 between 15:00 h and 22:00 h each (Figure 1).

### 2.8 Western blot

At PND56, one offspring rat was randomly selected from each litter and euthanized by cervical dislocation, and the hippocampal tissue was rapidly dissected on ice. Then, hippocampal tissues were homogenized in a protein lysis buffer containing protease and phosphatase inhibitors using an ultrasonic cell disruptor. Equal portions of protein (50 μg) were mounted onto Tris-glycine gels. Membranes were stored at 4°C and blocked with 4% BSA for 30 min. The membranes were then incubated overnight at 4°C with anti-GSK-3β (1:1,000), anti-β-catenin (1:5,000), and anti-Glyceraldehyde-3-phosphate dehydrogenase (GAPDH; 1:5,000). The membranes were then rinsed three times with TBST and incubated with anti-rabbit (1:5,000) secondary antibody for 1.5 h. The membranes were developed using a chemiluminescent gel imager, and the protein bands were subsequently analyzed for grayscale values using the Image J software.

### 2.9 Statistical analysis

SPSS26 and GraphPad Prism7 were used to analyze the data. Measurement data are expressed as the mean and standard deviation. After normality and homogeneity of variance tests were met, one-way ANOVA was used to compare multiple groups, and Tukey’s multiple comparisons were used for the post-test. After the two-factor design data and repeated measures data met tests for normality, homogeneity of variance, and “spherical symmetry,” differences between multiple groups were compared using two-way ANOVA and repeated measurement ANOVA. The two-sided test level α=0.05, *p* < 0.05, means the difference is statistically significant.

## 3 Results

### 3.1 Exposure to B[a]P delays the developmental node of the offspring

**Figure 2:**
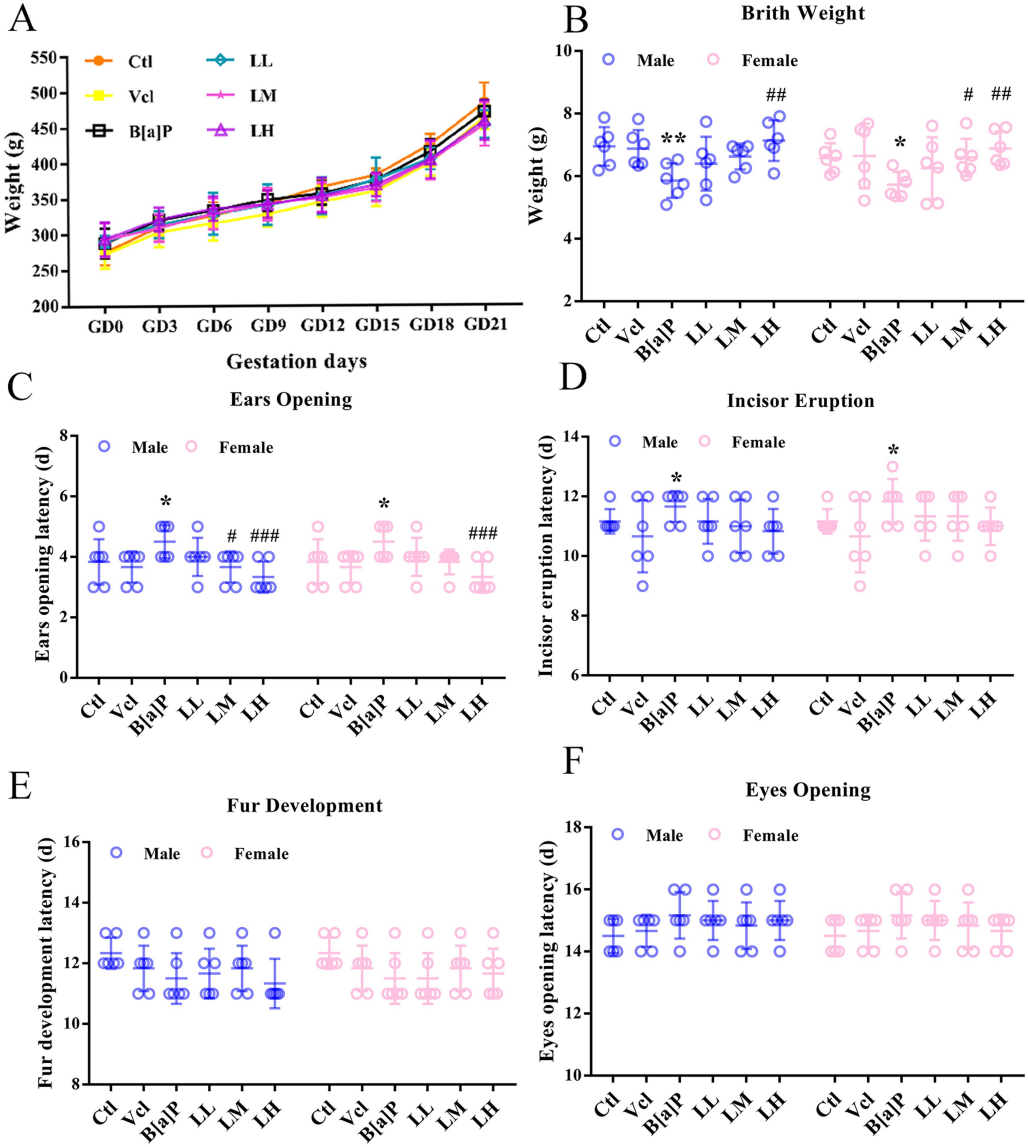
Effects of exposure to B[a]P on the developmental landmarks. **(A)** Body weight gain during gestation; **(B)** birth weight; **(C)** ear opening; **(D)** incior eruption; **(E)** fur development; **(F)** eyes opening. Values were expressed as Mean ± SD (*n* = 6); *p < 0.05, **p < 0.01 significantly different from control group; #*p* < 0.05, ##*p* < 0.01, ###*p* < 0.001 significantly different from B[a]P.

During pregnancy, there were no significant differences in the skin, fur, diet, water intake, and activity levels of pregnant rats in the B[a]P group compared to the control group. Moreover, B[a]P exposure did not affect the pregnant rats’ weight gain (one-way ANOVA: *F* = 0.341, *p* = 0.8811, Figure 2A), nor did it lead to premature delivery or dead fetuses. The number of litters and sex ratios of offspring rats in each group were normal, and no malformed offspring were observed. Developmental milestones are key developmental stages that children reach as they grow that mark their progress in physical functioning and cognitive abilities. In the present study, the effect of B[a]P exposure on birth weight, ear opening, and incisor eruption in offspring rats was demonstrated (two-way ANOVA: *F*_(5,60)_ = 4.881, *p* < 0.001; *F*_(5,60)_ = 5.397, *p* < 0.001; *F*_(5,60)_ = 2.442, *p* = 0.04). Analysis showed that the B[a]P group had a significantly reduced birth weight of the offspring rats (male: *q =* 2.796*, p =* 0.007, female: *q =* 2.187*, p =* 0.0327, Figure 2B) and delayed ear opening time (male: *q* = 2.49, *p* = 0.016, female*: q* = 2.49, *p* = 0.016, Figure 2C), and incisor eruption time (male: *q* = 2.153, *p* = 0.0354, female: *q* = 2.512, *p* = 0.015, Figure 2D). As the fetal mice grew, the fur and eye-opening time nodes after 10 days of birth were not delayed (Figures 2E, F).

### 3.2 Exposure to B[a]P decreases the sensitivity of neural reflex functions in offspring

**Figure 3:**
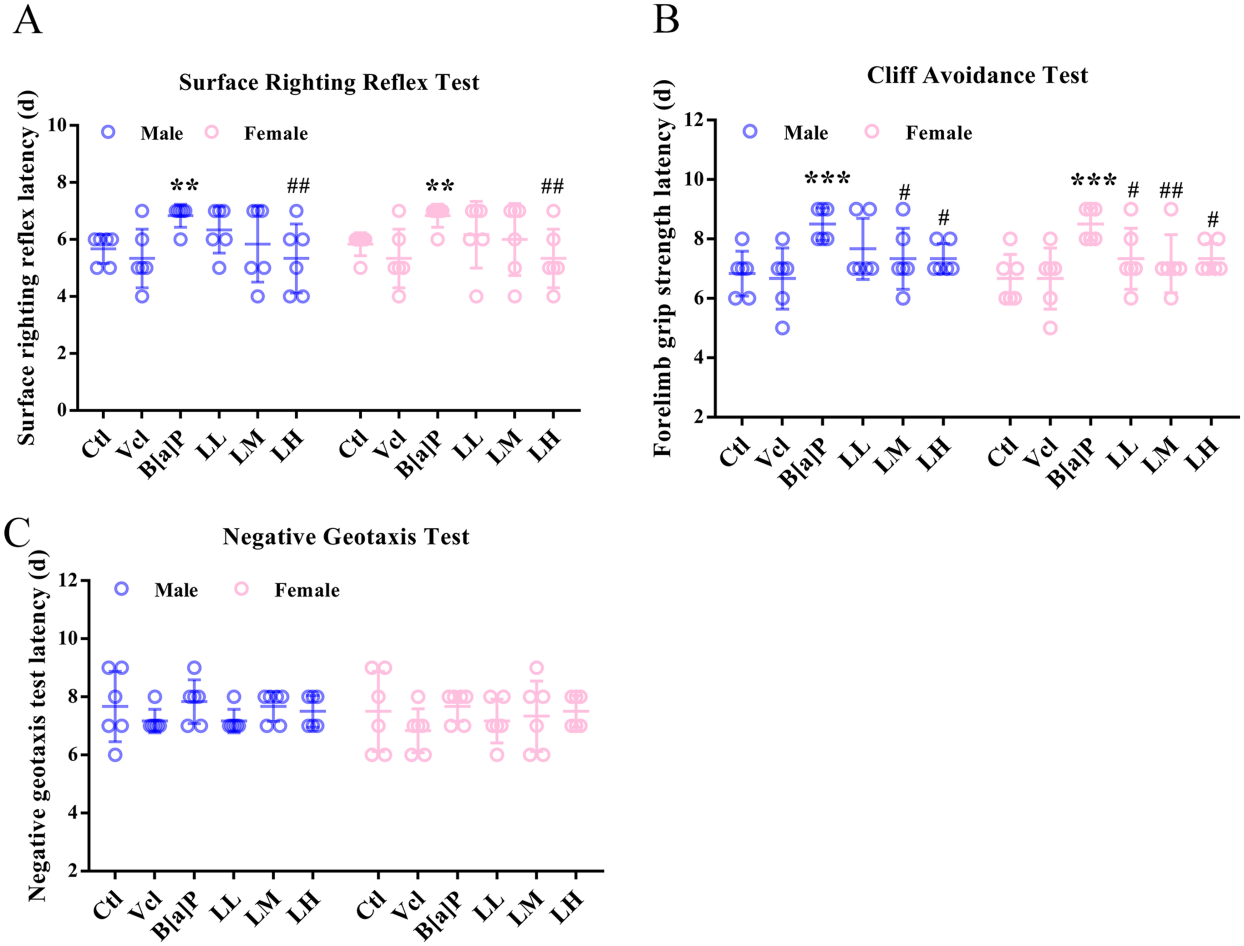
Effects of exposure to B[a]P on the neonatal sensory and motor development. **(A)** Surface righting reflex test; **(B)** cliff avoidance test; **(C)** negative geotaxis tests. Values were expressed as Mean ± SD (*n* = 6). ** *p* < 0.01, *** *p* < 0.001 means significantly different from control group; #*p* < 0.05, ##*p* < 0.01 means significantly different from B[a]P group.

The righting reaction, a test that evaluates early childhood neurodevelopment and motor skills, is important for understanding a child’s sensory integration and motor coordination. Similar neurodevelopmental reflex tests in animals can test for normal positional sensory function of vision, the vestibular system, and pressure receptors or tactile receptors in the limbs. The results showed that exposure to B[a]P during mid-pregnancy had a remarkable effect on the righting reflex and cliff aversion test (two-way ANOVA: *F*_(5,60)_ = 4.409, *p* = 0.002; *F*_(5,60)_ = 7.246, *p* < 0.001). Offspring rats in the B[a]P exposure group need longer to return to the normal four-foot landing position (male: *q* = 2.734, *p* = 0.008, female: *q* = 2.734, *p* = 0.008, Figure 3A) and to turn or retreat from the edge of the cliff (male: *q* = 3.737, *p* < 0.001, female: *q* = 3.737, *p* < 0.001, Figure 3B). Within the negative geotaxis test, it was found that there was no significant effect of B[a]P treatment (Figure 3C).

### 3.3 Exposure to B[a]P reduces neuronal dendritic complexity in the hippocampal CA1 region

**Figure 4:**
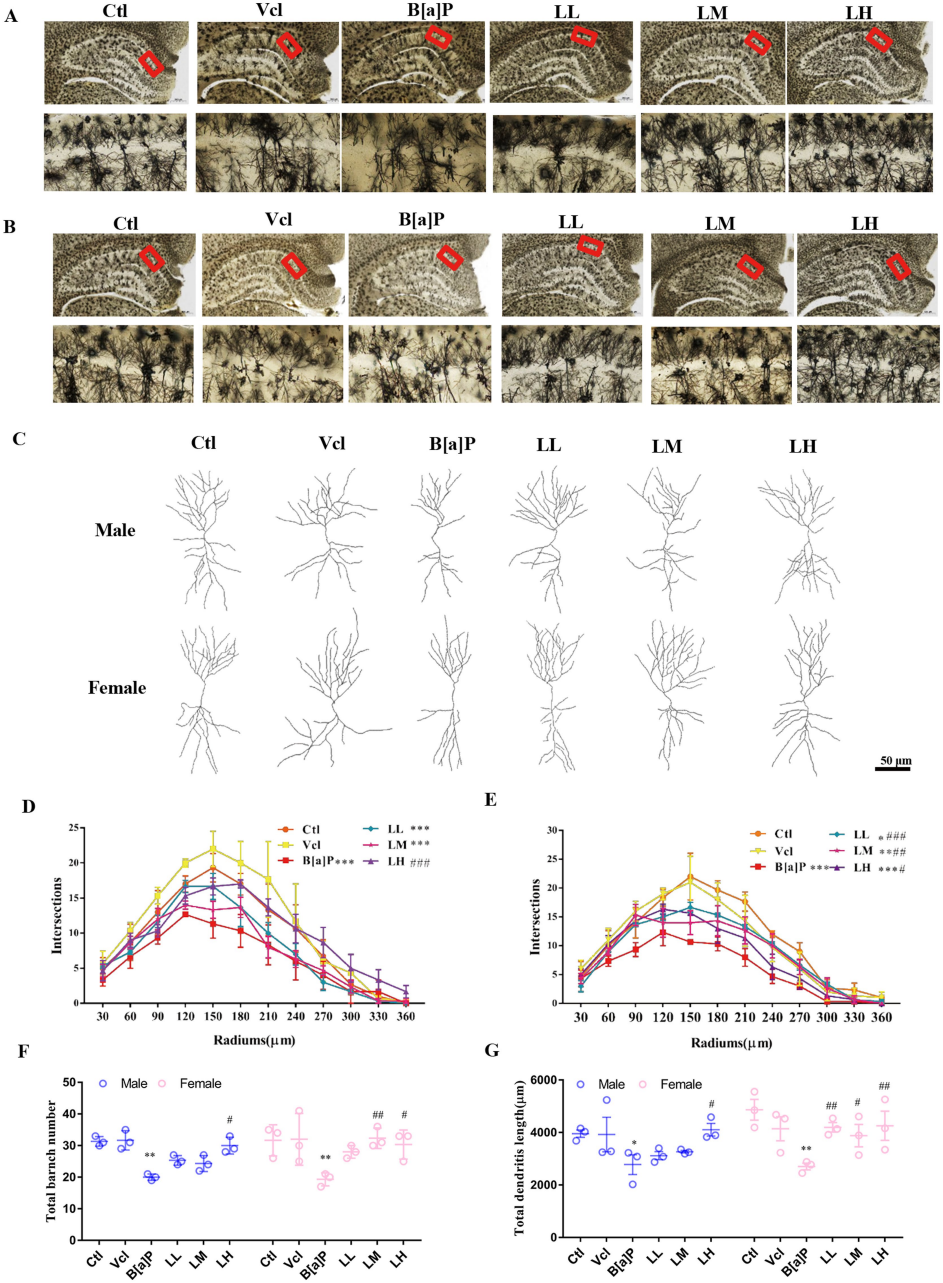
Effects of exposure to B[a]P on dendritic spines of hippocampal CA1 region by Golgi stain. Values were expressed as Mean ±SD (n = 3); **(A)** and **(B)** are neuronal morphologies in the hippocampal region of male and female rats, respectively. Bar = 500 μm, Bar = 50 μm; **(C)** is the examples of reconstructed Golgi-impregnated pyramidal neurons in the CA1 region; **(D)** and **(E)** are neuronal complexity in the CA1 region of the hippocampus in male and female rats, respectively; **(F)** is the total number of branches of neuronal dendrites in the CA1 region of the hippocampus; **(G)** is the total dendritic length of neuronal dendrites in the CA1 region of the hippocampus. *p < 0.05, **p < 0.01, ***p < 0.001 significantly different from control group. #*p* < 0.05, ##*p* < 0.01, ###*p* < 0.001 significantly different from B[a]P group.

To study CA1 pyramidal neuron morphology, we used the Golgi method to reconstruct 3D basal and apical dendritic trees from selected pyramidal neurons in three brains from each group (Figures 4A–C). The phenotype was also confirmed using Sholl analysis. The number of neuronal branches at each radius was significantly decreased in the B[a]P-exposed group in male offspring (two-way ANOVA: *F*_(5,144)_ = 11.23, *p* < 0.001, *q* = 6.053, *p* < 0.001, Figure 4D) and female offspring (two-way ANOVA: *F*_(5,144)_ = 13.95, *p* < 0.001, *q* = 9.26, *p* < 0.001) (Figure 4E). Pyramidal dendrites in the B[a]P-treated offspring rats were less branched (two-way ANOVA: *F*_(5,24)_ = 8.468, *p* < 0.001, male: *q* = 5.54, *p =* 0.008, female: *q* = 6.015*, p =* 0.003, Figure 4F), and total branch length was shorter than control group in the CA1 region (two-way ANOVA: *F*_(5,24)_ = 5.872, *p* < 0.001, male: *q* = 2.234, *p =* 0.035; female: *q* = 2.806, *p =* 0.0098, Figure 4G).

### 3.4 Exposure to B[a]P impairs episodic memory function in adult offspring

**Figure 5:**
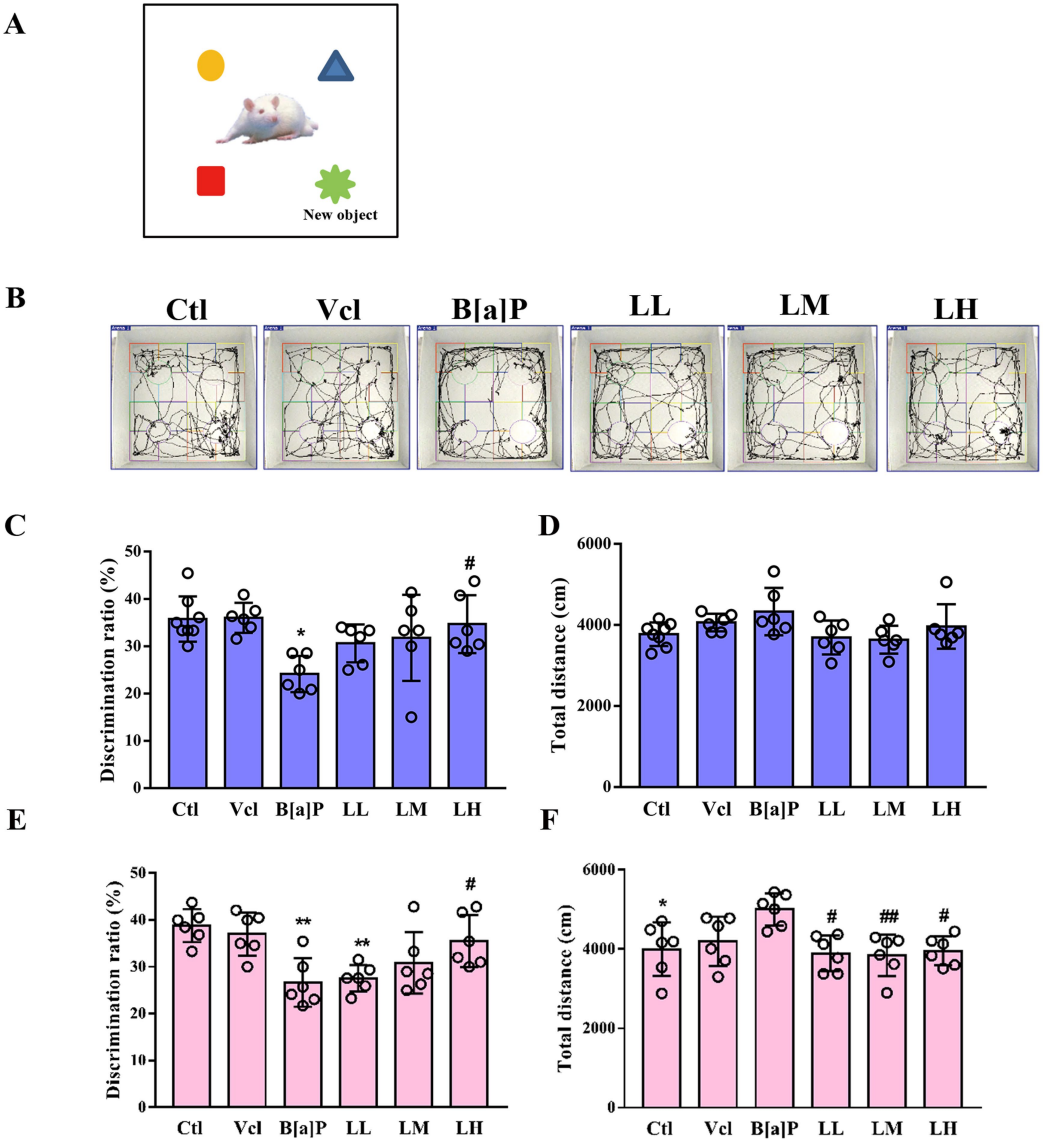
Effects of exposure to B[a]P on the learning and memory; **(A)** is the sketch map of novel object recognition test; **(B)** are the trajectories of offspring rats; **(C)** is the discrimination radio of novelty in male rats; **(D)** is the total distance traveled by male rats; **(E)** is the discrimination radio of novelty in female rats; **(F)** is the total distance traveled by female rats. Values were expressed as Mean ± SD (*n* = 6). *p < 0.05; **p < 0.01, means significantly different from control group; #*p* < 0.05, ##*p* < 0.01 means significantly different from B[a]P group.

To assess animals’ episodic memory (EM) function, particularly their recognition memory and ability to retain information about novel objects, we conducted the novel object recognition (NOR) test. The schematic diagram and trajectory plots of the novel object recognition test are shown in Figures 5A,B, respectively. In our study, male offspring in the B[a]P-exposed group had a significantly lower novel object recognition rate than the control group (one-way ANOVA: *F*_(5,30)_ = 3.799, *p* = 0.009, *q* = 5.018, *p* = 0.016, Figure 5C). Meanwhile, the total distance moved between male groups showed no significant difference (*F*_(5,30)_ = 2.294, *p* = 0.072, Figure 5D). In female offspring, we also observed a decline in the ability to recognize novel objects in the B[a]P-exposed group (one-way ANOVA: *F*_(5,30)_ = 6.586, *p* < 0.001, *q* = 6.104*, p* = 0.002, Figure 5E). However, we also noticed that female offspring in the B[a]P-exposed group exhibited a significant increase in total traveled distance (*F*_(5,30)_ = 4.211, *p* = 0.005, *q* = 4.729*, p* = 0.025, Figure 5F).

### 3.5 Exposure to B[a]P impairs spatial memory function in adult male offspring

**Figure 6:**
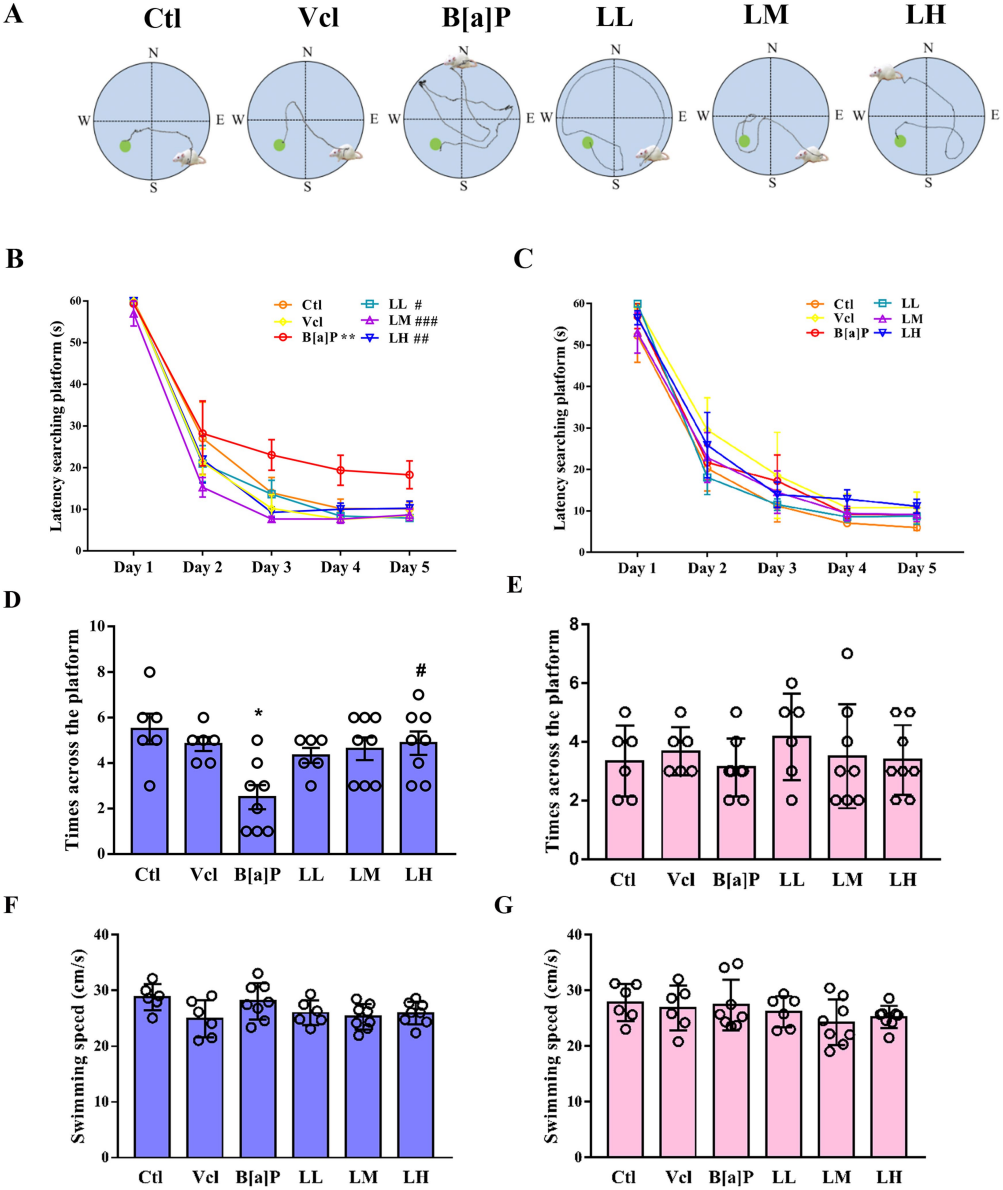
Effects of exposure to B[a]P on the learning and memory; **(A)** is the trajectories of offspring rats; **(B)** is the time when male rats searched for the hidden platform; **(C)** is the times when female rats searched for the hidden platform; **(D)** is the number of times male rats crossed the hidden platform on day 6; **(E)** the number of times female rats crossed the hidden platform on day 6; **(F)** the swimming speed of male rats; **(G)** the swimming speed of female rats. Values were expressed as Mean ± SD (*n* = 6–8). **p < 0.05 significantly different from control group; #*p* < 0.05, ##*p* < 0.01, ###*p* < 0.001 significantly different from B[a]P group.

To assess spatial memory function in animals, especially in hippocampus-dependent memory tasks, the Morris water maze (MWM) experiment is used. The schematic diagram of the water maze test is illustrated in Figure 6A. In the training phase, male offspring exposed to B[a]P need more time to find hidden platforms (repeated measures ANOVA, *F*_(5,36)_ = 7.697, *p* < 0.001). However, this impairment of memory function is independent of time (treatment × time: *F*_(20,160)_ = 0.612, *p* = 0.898). Subsequently, main effect analyses displayed that rats exposed to B[a]P took more time to find hidden platforms on day 5 than controls (*q* = 5.673*, p* = 0.004, Figure 6B). In the probe trial test of the water maze, the times across the platform of rats exposed to B[a]P were significantly decreased (one-way ANOVA, *F*_(5,36)_ = 3.7, *p* = 0.009; *q* = 5.107*, p* = 0.012, Figure 6D). However, in female offspring, we did not observe a decline in spatial memory function in the B[a]P group (*F*_(5,36)_ = 1.407, *p* = 0.245; *F*_(5,36)_ = 0.5079, *p* = 0.7684, Figures 6C,E). To rule out the interference of impaired motor function in offspring, swimming speed during the test on day 6 was further analyzed in male and female offspring. No significant differences were found between groups (male: *F*_(5,36)_ = 2.355, *p* = 0.060; female: *F*_(5,36)_ = 1.045, *p* = 0.407, Figures 6F,H).

### 3.6 Exposure to B[a]P inhibits β-catenin translocation to the nucleus in the Wnt/β-catenin pathway

**Figure 7:**
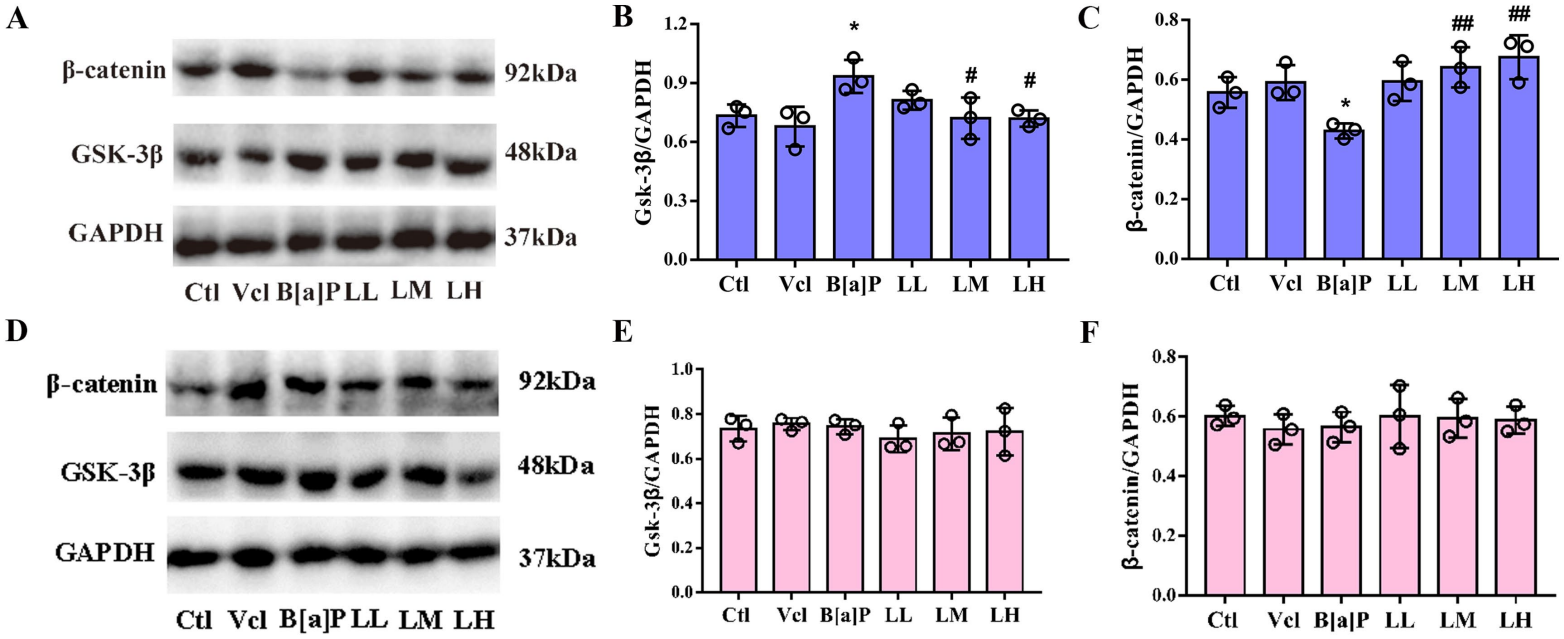
Effects of exposure to B[a]P on GSK-3β/β-catenin pathway; **(A)** is immunohistochemical protein maps of the hippocampus of male rats; **(B)** is hippocampal GSK-3β protein expression in male rats; **(C)** is β-catenin protein expression in male rats; **(D)** is immunohistochemical protein maps of the hippocampus of female rats; **(E)** is hippocampal GSK-3β protein expression in female rats; **(F)** is β-catenin protein expression in female rats. Values were expressed as Mean ± SD (*n* = 3). *p < 0.05, significantly different from vehicle group; #*p* < 0.05, ##*p* < 0.01 significantly different from B[a]P group.

To better examine the relationship between the Wnt/β-catenin pathway in hippocampal tissue and offspring neurological impairment due to B[a]P exposure during pregnancy, we examined the activity of key Wnt/β-catenin pathway proteins. In PND56, compared to the control group, the expression of GSK-3β protein in male hippocampus tissue of the B[a]P exposure group was significantly increased (one-way ANOVA: *F*_(5,12)_ = 4.364, *p* = 0.017, Tukey’s test: *q* = 5.736, *p* = 0.015), and the expression of downstream β-catenin protein was significantly decreased (one-way ANOVA: *F*_(5,12)_ = 6.307, *p* = 0.004, Tukey’s test: *q* = 4.766, *p* = 0.049, Figures 7A–C). However, in female offspring, no significant changes in the protein levels of the key proteins β-catenin and GSK-3β were detected between groups (*F*_(5,12)_ = 0.2702, *p* = 0.921, *F*_(5,12)_ = 0.399, *p* = 0.840, Figures 7D–F).

### 3.7 All of these indicators of B[a]P alteration can be reversed by LiCl

Lithium chloride, an agonist of the Wnt/β-catenin pathway, specifically binds GSK-3β and inactivates phosphorylation (Gould et al., 2007). In the present study, we continued to observe the ameliorative effects of LiCl at the individual, cellular, and protein levels.

We administered LiCl at 10 mg/kg (LiCl-L), 20 mg/kg (LiCl-M), and 40 mg/kg (LiCl-H) alone to pregnant rats. The weight changes of LiCl group rats during pregnancy showed no statistical difference compared to the control group (*F* = 1.452, *p* = 0.2857, Supplementary Figure 1A). Among the offspring, only those in the LiCl-H group had an earlier incisor eruption time node (male: *F* = 3.778, *p* = 0.027, *q* = 4.082, *p* = 0.042; female: *F* = 4.31, *p* = 0.017, *q* = 4.983, *p* = 0.011, Supplementary Figure 1D). In the LiCl-M group, the time node of hair growth in offspring was advanced too (male: *F* = 3.049, *p* = 0.05, *q* = 4.191, *p* = 0.0356; female: *F* = 3.049, *p* = 0.05, *q* = 4.191, *p* = 0.0356, Supplementary Figure 1E). Compared to the control group, the neurodevelopment reflexes and learning memory functions in the offspring exhibited no significant differences (Supplementary Figures 1G–I, 2). In male offspring, the dendritic complexity in the hippocampal CA1 region is significantly increased (*q* = 4.062, *p* = 0.0249, Supplementary Figure 3D). LiCl alone does not affect the protein expression levels of GSK-3β and β-catenin in offspring (Supplementary Figure 4).

At the level of individuals, LiCl (LM and LH groups) can restore the birth weight of offspring rats (male: *q* = 3.278, *p* = 0.002; female: *q* = 2.237, *p* < 0.029, *q* = 2.952, *p* < 0.005, Figure 2B) and reverse B[a]P-induced delay in ear opening time (male: *q* = 2.49, *p* = 0.016, *q* = 3.486, *p* = 0.001; female: *q* = 3.486, *p* < 0.001, Figure 2C). The time of righting reflex was significantly earlier in the high-dose LiCl intervention group compared to the B[a]P group (male: *q* = 2.734, *p* = 0.0082; female: *q* = 2.734, *p* = 0.0082, Figure 3A). LiCl (LM and LH groups) intervention significantly improved their sensory ability (male: *q* = 2.378, *p* = 0.021, *q* = 4.756, *p* = 0.016; female: *q* = 2.378, *p* = 0.021, *q* = 2.717, *p* = 0.009, *q* = 4.789*, p* = 0.019, Figure 3B). In addition, LiCl (LH group) intervention significantly restored episodic memory function and spatial memory function in adult male offspring (*q* = 4.518, *p* = 0.037, Figure 5C; *q* = 5.848*, p* = 0.003, Figure 6B; *q* = 4.796*, p* = 0.022, Figure 6D). In adult female offspring, LiCl (LH group) intervention significantly restored episodic memory function (*q* = 4.436, *p* = 0.04, Figure 5E).

At the neuronal cellular level, LiCl showed a similar ameliorating effect, significantly increasing dendritic complexity in the CA1 region of the hippocampus (male: *q* = 6.311, *p* < 0.001, Figure 4D; female: *q* = 6.211, *p* < 0.001, *q* = 5.533, *p* = 0.002, *q* = 4.743, *p* = 0.013, Figure 4E). Furthermore, LiCl intervention significantly increased the branch number (male: *q* = 4.748*, p* = 0.028, female: *q* = 6.173, *p* = 0.003, *q* = 5.223, *p* = 0.013, Figure 4F) and length (male: *q* = 2.585*, p* = 0.016, female: *q* = 2.91, *p* = 0.008, *q* = 2.291, *p* = 0.031, *q* = 3.023, *p* = 0.006, Figure 4G) in CA1 region of the hippocampus.

On the protein level, LiCl (LM and LH groups) significantly reversed the increase of GSK-3β and the decrease of β-catenin caused by B[a]P in male offspring (*q* = 4.785, *p* = 0.048, *q* = 4.823, *p* = 0.046; *q* = 6.252, *p* = 0.008, *q* = 7.241, *p* = 0.003, Figure 7B,C).

## 4 Discussion

B[a]P, highly lipid-soluble, crosses the placental barrier, enters the fetal brain, and causes damage due to the brain’s characteristics (Topinka et al., 2009). It accumulates in brain tissues and induces neurodevelopmental toxicity (Bao et al., 2019; Olasehinde and Olaniran, 2022). As a neurotoxicant, B[a]P can lead to unresponsiveness and learning/memory deficits through specific nervous system damage. These findings have been validated in various models (Narvaes and Furini, 2022; Ramaiah et al., 2023).

In this experiment, we found that mid-gestation exposure to B[a]P not only resulted in delayed developmental milestones and reduced sensitivity to neural reflex functions in the early postnatal period but also impaired the learning and memory functions of the offspring in adulthood. Current research suggests that *in utero* exposure to B[a]P (25 mg/kg) may cause lasting neural and behavioral impairments. These issues might not be evident during embryonic development but can emerge in early postnatal life and persist into young adulthood, with adverse effects potentially worsening with age. A study by Chengzhi Chen et al. also showed a long-term potential developmental toxicity effect after B[a]P exposure. B[a]P administered early in the postnatal period had no significant effect on incisor eruption, hair growth, and eye-opening in the offspring, but it reduced adolescent and adult body weight (Chen et al., 2012).

In this study, ear opening and budding were significantly delayed in the B[a]P group, while hair growth and eye-opening were unaffected. This may be because the damaging effects of B[a]P are reduced after the second rapid development of the fetal brain or are specific only to ear opening and tooth sprouting (Lebel and Deoni, 2018). The exact reasons and mechanisms require further exploration. Similarly, in the B[a]P-exposed group, offspring rats took longer to perform the righting reflex and to turn or retreat from the cliff’s edge. This may stem from delayed skeletal muscle development due to lower birth weight, weakening the muscle strength needed for these motor behaviors (Li et al., 2017). However, by postnatal day 10, the B[a]P-exposed group’s body weight and muscle strength recovery might offset early deficits, normalizing negative geotaxis test performance.

In this study, both novelty recognition and water maze tests showed a significant reduction in the learning and memory functions of male rat offspring exposed to B[a]P during pregnancy, an effect that persists into adulthood. Consistently, previous experiments demonstrated that oral B[a]P exposure on postnatal days P5–11 impaired adult rats’ learning and memory (Maciel et al., 2014). However, our study found no adult spatial learning and memory deficits in female B[a]P group offspring. Moreover, it is speculated that the female offspring may be affected by the estrogen level in the body, which can cause inconsistent behavioral test results (Choleris et al., 2011). Further experiments are needed for verification.

B[a]P exposure during adolescence or adulthood impairs rodents’ learning and memory (Patel et al., 2016). This may be associated with neurotransmitters, synaptic plasticity, dendritic spine density, and the structural integrity of hippocampal neurons (Zola-Morgan et al., 1989). Our study finds that mid-pregnancy B[a]P exposure may affect the complexity of hippocampal CA1 neuronal structures. This is consistent with past research showing that B[a]P-exposed rats may suffer severe neuronal damage, including changes in neuron number, volume, dendritic structure, dendritic spine shape, and synaptic function (Lyu et al., 2020). In our study, the B[a]P-exposed group’s neurons had reduced dendritic branches and total length. This may impair the hippocampal neural network’s information integration, resulting in spatial navigation and memory formation defects (Mehder et al., 2020; Yousef et al., 2019).

**Figure 8:**
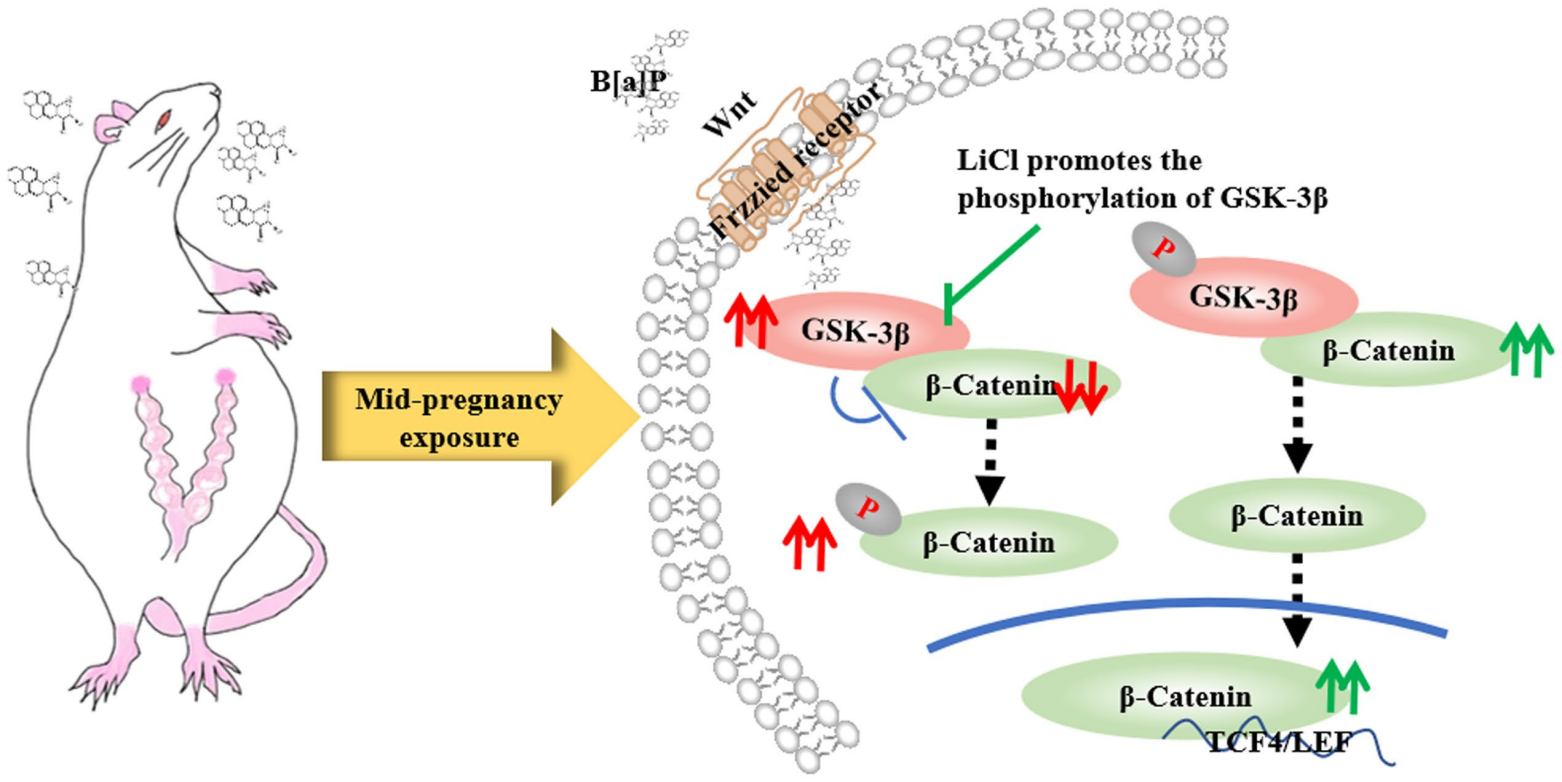
LiCl’s regulatory action on the GSK-3β/β-catenin axis.

The Wnt/β-catenin signaling pathway regulates hippocampal neuron proliferation, differentiation, migration, and synaptic plasticity (Fan et al., 2022). Since neurogenesis and synaptic plasticity are closely associated with learning and memory functions, we hypothesize that this signaling pathway is involved in multiple fetal neurodevelopmental processes. In our study, exposure to B[a]P during mid-pregnancy was found to significantly increase the expression of GSK-3β in the hippocampus of their male offspring while decreasing the expression of β-catenin in the same region. This may be because B[a]P exposure disrupts Wnt protein activation and prevents its binding to receptors on the cell membrane. Consequently, it activates GSK-3β protein sites in the cytoplasm, increasing the degradation complex containing GSK-3β. This leads to further phosphorylation and degradation of downstream β-catenin protein, ultimately reducing its migration and accumulation in the nucleus (Figure 8). Thus, it cannot initiate downstream gene transcription for biological effects. This aligns with prior findings that sustaining GSK-3β’s activated state impairs learning, memory, and cognitive abilities in various experimental animal behavioral models (Hui et al., 2018; Dewachter et al., 2009). Previous studies have indicated that reduced β-catenin expression can disrupt brain and spinal cord tissue development, preventing normal neuronal development (Gould et al., 2007). This is consistent with the decreased neuronal complexity observed in the hippocampal CA1 region of the B[a]P group in our study.

LiCl is an inhibitor of the GSK-3β protein, which has the effects of neurotrophic, neuron protection, and promotion of nerve regeneration (Hui et al., 2018). In our experiment, LiCl restored the birth weight, ear opening, and incisor eruption of offspring rats. It also significantly repaired the motor, sensory, and memory impairments induced by B[a]P. Further studies revealed that LiCl treatment significantly ameliorated the B[a]P-induced decreases in dendritic complexity, total dendritic branching, and total dendritic length in the hippocampal CA1 region. This may be related to lithium chloride’s function in promoting neuronal growth. In this study, LiCl intervention effectively elevates β-catenin protein levels. This is because LiCl enhances the phosphorylation of GSK-3β at Ser9 (p-GSK-3β), further inhibiting GSK-3β enzymatic function and reducing the amount of degradation complex containing GSK-3β, which in turn decreases the phosphorylation of β-catenin in the cytoplasm (Junde et al., 2023). Consequently, the level of cytoplasmic β-catenin protein increases, and its migration to the nucleus is enhanced, promoting the growth and differentiation of neural stem cells (Oliva et al., 2013) (Figure 8).

## 5 Limitations

Although this study tracked and analyzed different periods after B[a]P exposure, there are some limitations that need to be mentioned. Considering the uncertainty of the survival of the offspring at the later stage after birth, the animals were not euthanized at birth for Golgi staining and Western blot experiments to ensure the behavioral experiments were carried out smoothly.

## 6 Conclusion

In summary, we found that B[a]P exposure in mid-gestation pregnancy may lead to developmental landmark delay, decreased neuromotor reflex function, and impaired learning and memory ability in offspring by downregulating the Wnt/β-catenin signaling pathway. LiCl, as an activator of the Wnt/β-catenin signaling pathway, can reverse the harmful effects of B[a]P.

## Data Availability

The original contributions presented in the study are included in the article/Supplementary material, further inquiries can be directed to the corresponding author.
