# Supplementary figures and images for: Maternal benzo[a]pyrene exposure during critical gestational periods impairs offspring neurological development in rats: a mechanistic study of the Wnt/β-catenin signaling pathway
Source: Front Behav Neurosci. 2025 Apr 24;19:1571122. doi: 10.3389/fnbeh.2025.1571122 (PMC12058798; doi:10.3389/fnbeh.2025.1571122)

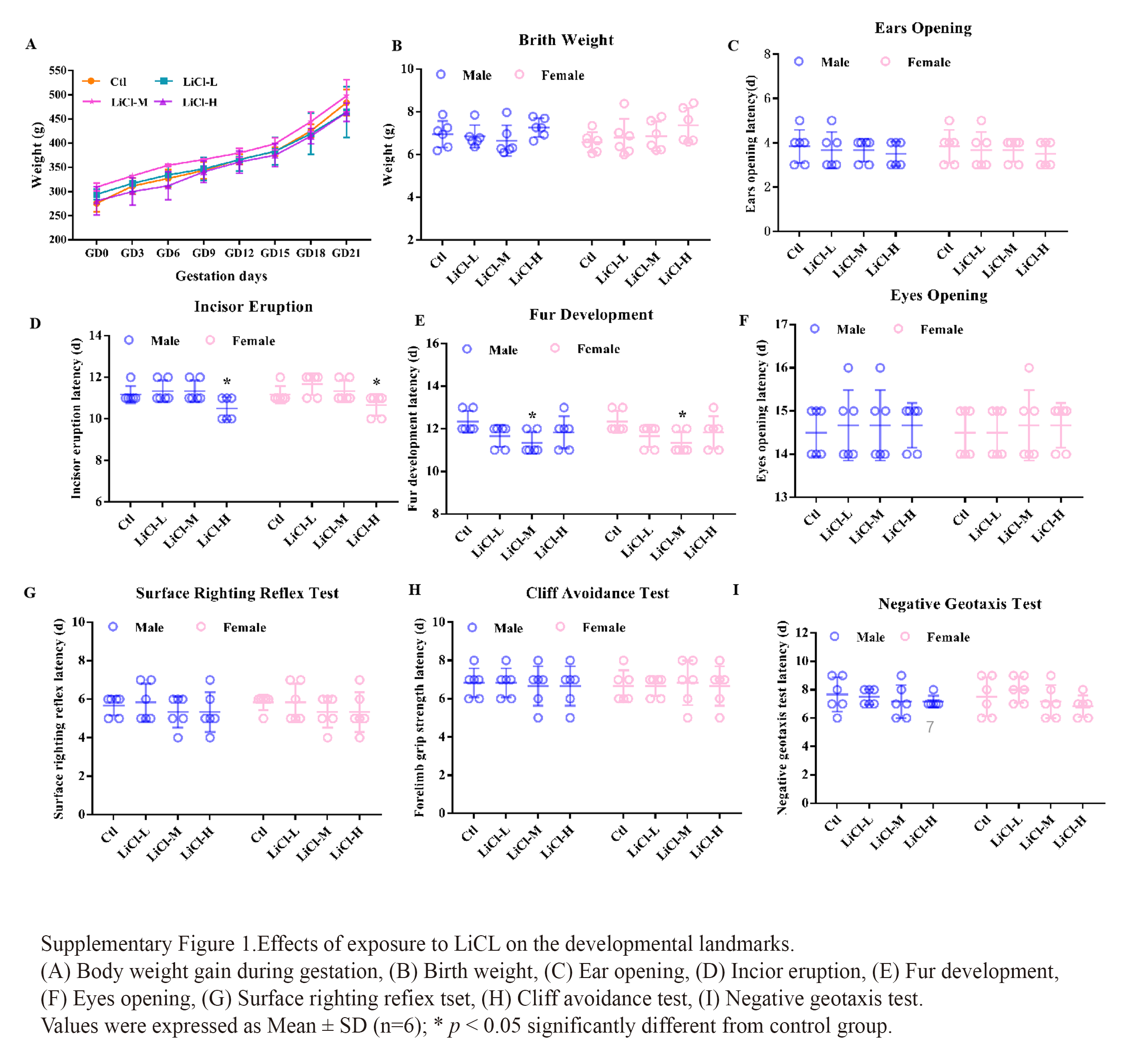

Supplement: Supplementary file 1 [file Image_1.tif]

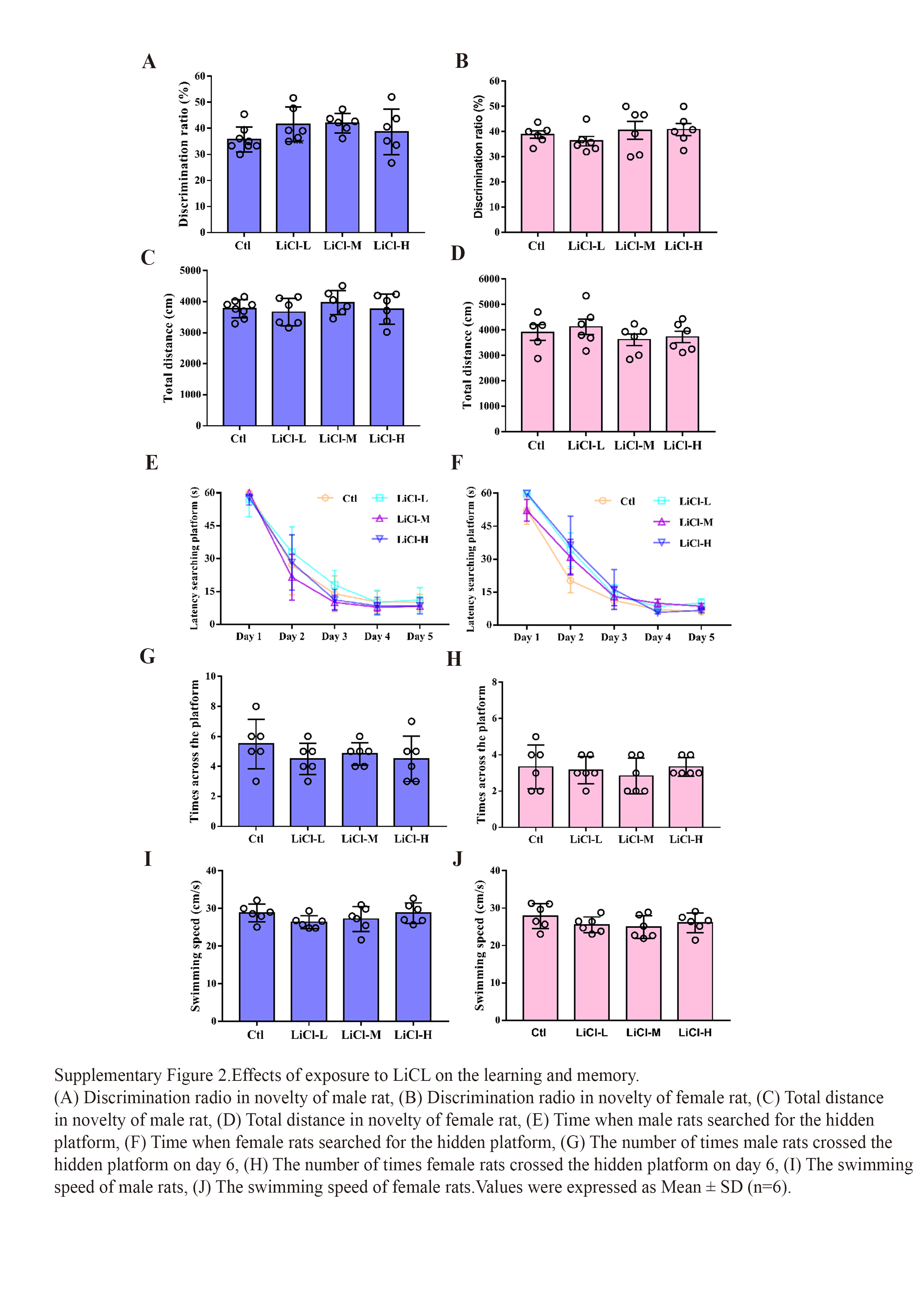

Supplement: Supplementary file 2 [file Image_2.tif]

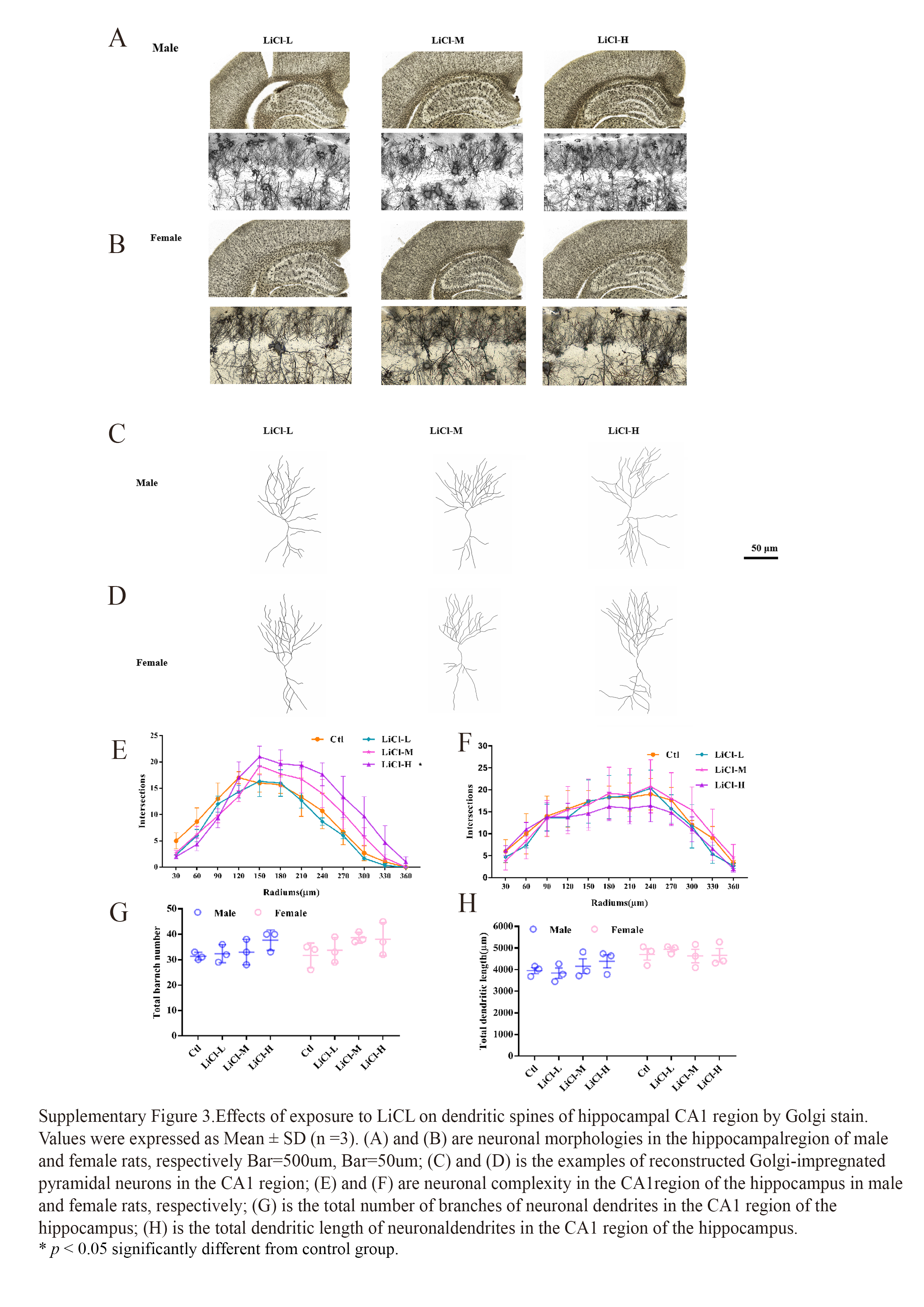

Supplement: Supplementary file 3 [file Image_3.tif]

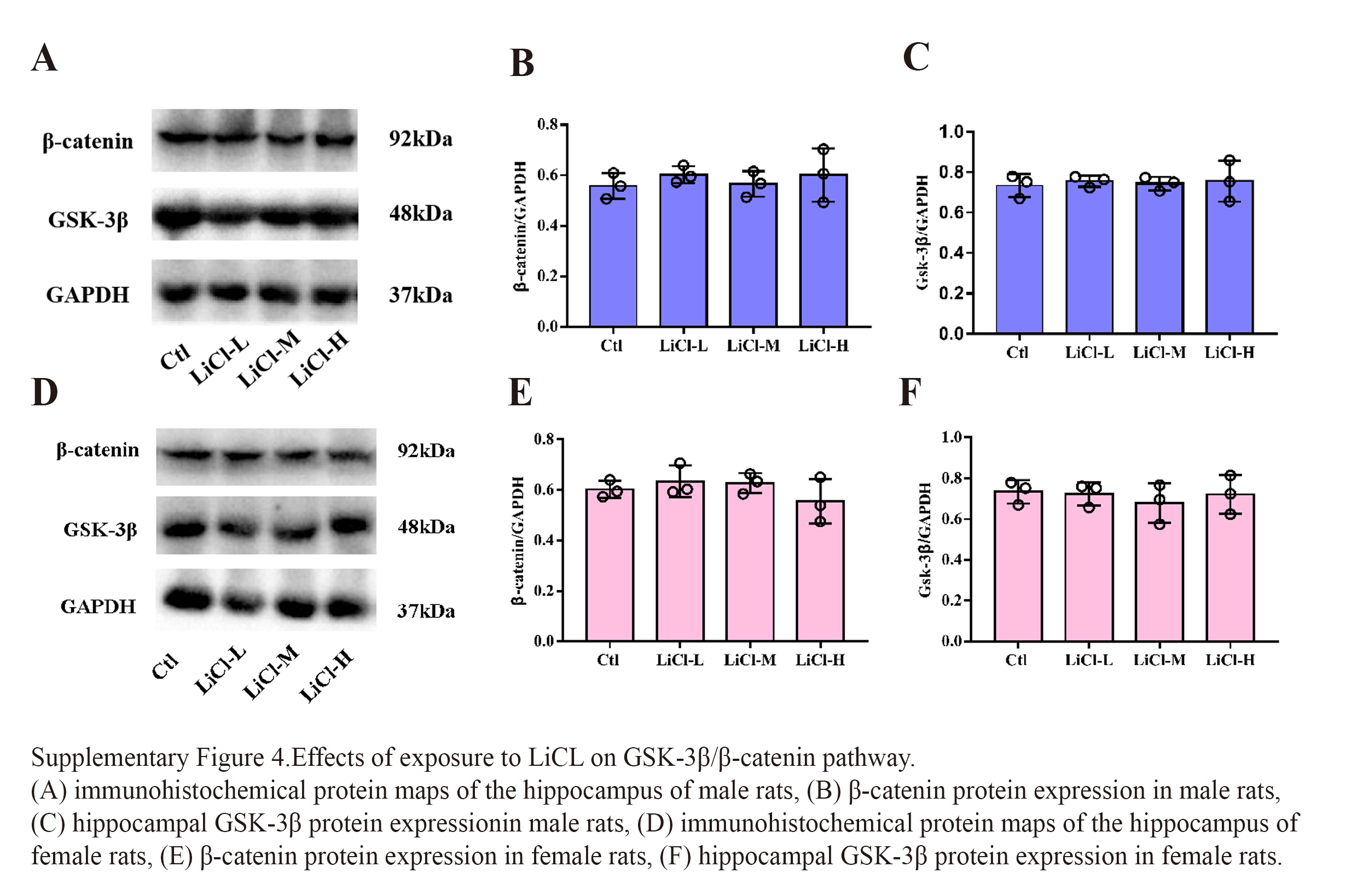

Supplement: Supplementary file 4 [file Image_4.tif]
